# Cross-Lagged Panel Analyses of Reciprocal Effects of Social Isolation, Perceived Loneliness, and Solitary Activity

**DOI:** 10.1093/geroni/igaa057.308

**Published:** 2020-12-16

**Authors:** Ke Li, Fengyan Tang

**Affiliations:** University of Pittsburgh, Pittsburgh, Pennsylvania, United States

## Abstract

Social isolation and perceived loneliness are major issues as they may place older adults at greater risks for health problems. The objective status of social isolation and the subjective perception of loneliness may have distinct meanings, and their longitudinal reciprocal relationship remains unclear. The purposes of this study were to examine the reciprocal effects of social isolation and loneliness among U.S. adults aged 50 and above and to explore the moderating effect of solitary activities by using the data from three waves of the Health and Retirement Study (HRS) collected in the year 2008, 2012 and 2016. The index of social isolation was created by summing five commonly used indicators, including marital status, living arrangement, and three types of social contact. Loneliness was assessed by a summary score of 11 items. Solitary activities included 13 activities with limited or no social interaction. The results estimated by the cross-lagged effects model showed positive reciprocal relationship of social isolation and perceived loneliness across waves: respondents with a higher level of social isolation were predicted to have increased loneliness, and more perceived loneliness was significantly associated with a higher level of social isolation in the following waves. The results also indicated that solitary activity had a direct effect on decreasing loneliness. This study improves the understanding of reciprocal effects of social isolation and perceived loneliness over years and indicates that practice needs to address the issues of social isolation and perceived loneliness at the early stage and provide more opportunities of solitary activities.

